# T1 Erector Spinae Plane Block Catheter As a Novel Treatment Modality for Pancoast Tumor Pain

**DOI:** 10.7759/cureus.6092

**Published:** 2019-11-07

**Authors:** Hari K Kalagara, Paige Deichmann, Brandon Brooks, Peter Nagi, Promil Kukreja

**Affiliations:** 1 Anesthesiology and Perioperative Medicine, University of Alabama, Birmingham, USA; 2 Anesthesiology, University of Alabama, Birmingham, USA

**Keywords:** erector spinae plane block, opioid consumption, neuropathic pain, cancer pain, pain management, analgesia

## Abstract

Pancoast tumors are non-small cell lung tumors, which can invade the ribs, vertebrae, sympathetic ganglia and brachial plexus. In this study, a patient with right-sided Pancoast tumor presented with intractable chronic pain on the right neck, upper extremity and chest wall. The chronic pain associated with Pancoast tumor, which was difficult to treat with opioids and other medications, was effectively treated with a high-thoracic erector spinae plane block (ESPB). Prolonged analgesia was provided with an ESP catheter to wean the patient from opioids. This case report provides an example where the novel interfacial ESP block can provide pain relief in challenging situations such as lung malignancies involving deeper structures and extensive areas of pain.

## Introduction

The erector spinae plane block (ESPB) is an interfascial plane block where local anesthetic (LA) is injected between the erector spine muscle and the transverse process. In 1922, Gaston Labat described similar approaches to block spinal nerves in relation to the transverse process at cervical, thoracic and lumbar vertebral levels. The ESPB was initially described to relieve chronic pain from metastatic disease and nonunion of rib fractures [1]. The ESPB has been shown to be effective in improving perioperative pain control for thoracic procedures, mastectomies, minimally invasive mitral valve and aortic valve surgery and video-assisted thoracic surgery (VATS) [2-10]. ESPB is also effective in improving postoperative pain control for abdominal, hip and pelvic surgeries, including caesarean section [11-17].

While the majority of case reports display the effectiveness of ESPB for perioperative analgesia, we present a unique case report of the successful use of ESPB for treating chronic pain due to Pancoast tumor. Initial cadaver studies revealed spread to the dorsal and ventral spinal nerve roots, and radiocontrast injection in patients confirms the spread of the injectate, resulting from the thoracic ESPB injection, into the thoracic paravertebral space across more than five intervertebral levels [1,18]. This extensive craniocaudal spread and analgesia from ESPB to more dermatomes from previous case reports enabled us to utilize this block in this patient to treat the severe pain affecting his right neck, shoulder, arm and chest wall.

## Case presentation

**Figure 1:**
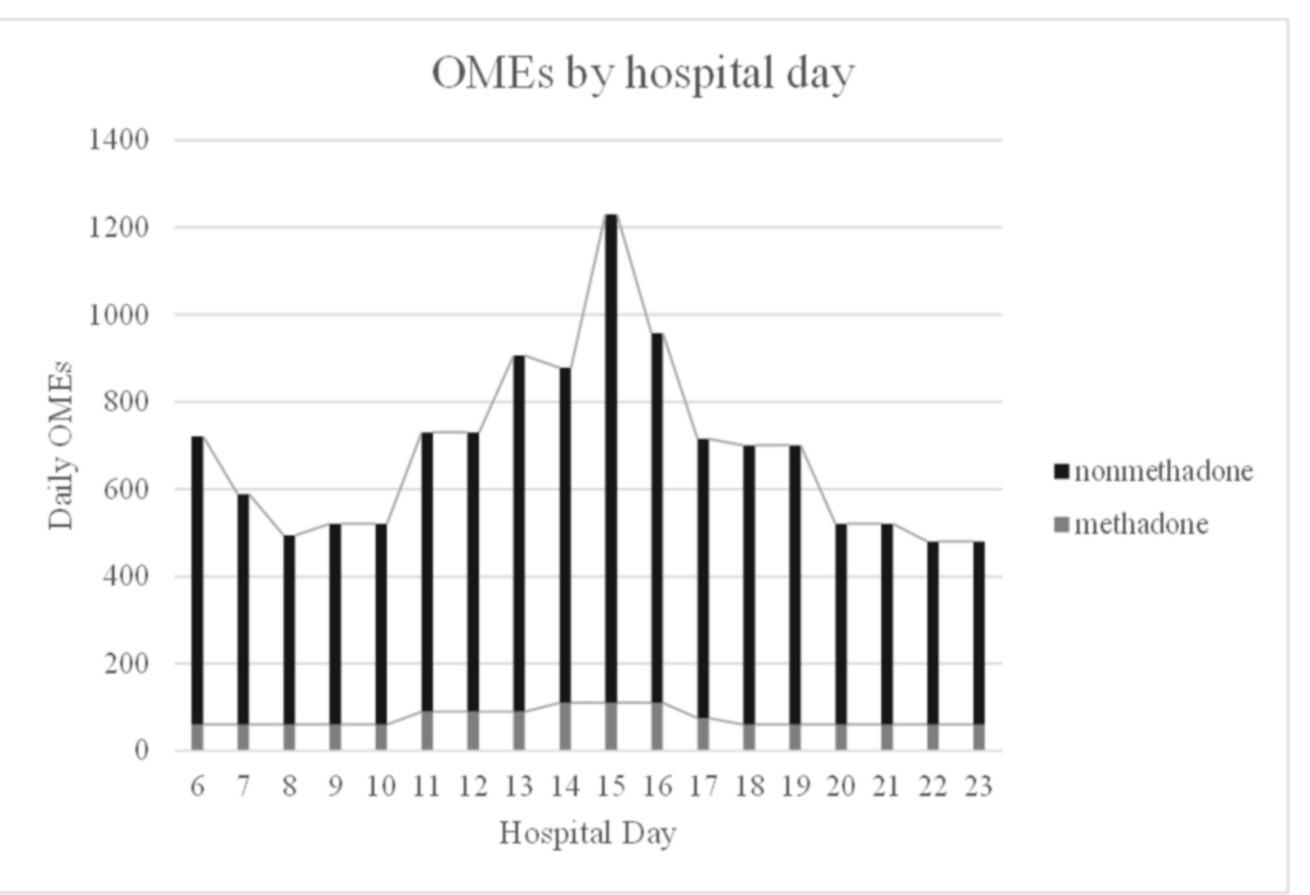
Daily OME dose of methadone and non-methadone opioids before and after ESP block catheter on day 16 OME, oral morphine equivalent; ESP, erector spinae plane

Written informed consent was provided by the patient for the procedure. A 55-year-old male of weight 66 kg and height 178 cm (body mass index [BMI], 20.8 kg/m^2^), with stage IV non-small cell lung cancer, presented to the emergency department with right upper extremity, neck, chest wall and abdominal pain, nausea, and non-bloody emesis. His lung cancer was previously treated with radiation therapy as well as chemotherapy with carboplatin, paclitaxel, and, most recently, pembrolizumab. At the time of presentation, the right apical lung mass measured 10.1 x 5.9 cm (Figure 1).

Imaging showed evidence of supraclavicular adenopathy as well as a brain metastasis to the occipital lobe, measuring 9 x 5 mm. The right Pancoast tumor invaded the right-sided brachial plexus and chest wall and eroded into the first and second ribs. As a result of tumor invasion, he developed chronic right upper extremity, neck and chest wall pain. His outpatient pain regimen included transdermal fentanyl 100 mcg q72hours, oral hydromorphone 16 mg QID PRN, and methadone 15 mg TID. The patient underwent cervical epidural steroid injections at pain clinics in the past with transient relief.

Upon admission to the hospital, his pain regimen was transitioned from oral to intravenous agents due to his inability to tolerate oral intake in the setting of ongoing emesis. He was started on a fentanyl infusion and his home methadone regimen was converted to intravenous dosing Over the following days, his bowel regimen was medically optimized, and his nausea resolved with improved bowel function. He was transitioned back to oral opioids; however, he continued to complain of intractable pain. He described this as sharp, burning pain localized to his right neck and shoulder with radiation down to his elbow, forearm and upper anterior chest wall. He characterized the pain as 9/10 in severity at baseline on a numeric rating scale (NRS) and 10/10 with activity. After a week's stay in the hospital, his daily methadone requirement had increased to 110 mg, a 244% increase from his home dose. On hospital day 16, the pain management service was consulted for his right-sided severe pain that had not improved despite the escalation of medical therapy with opioid analgesia (Table 1).

**Table 1:** Comparison of OME opioid usage of methadone and other opioids pre and post ESP catheter placement (day 16) OME, oral morphine equivalent; ESP, erector spinae plane

Hospital Day	24 hour OME, mg (excluding methadone)	Methadone dose (mg PO)	
6	661	60	
7	529	60	
8	434	60	
9	460	60	
10	460	60	
11	640	90	
12	640	90	
13	816	90	
14	768	110	
15	1120	110	
16 *catheter placed*	848	110	
17	640	75	
18	640	60	
19	640	60	
20	460	60	
21	460	60	
22	420	60	
23	420	60	

Because of the severity of his pain, anatomically wide pain distribution and response to a prior interventional procedure (cervical epidural steroid injection), we elected to attempt a regional anesthetic technique with the hopes of weaning down his daily opioid requirements. The risks and benefits of an infraclavicular brachial plexus nerve catheter were discussed. After we reviewed prior imaging and assessed with bedside ultrasound, the infraclavicular block was ruled out due to the extent of tumor invasion and its proximity to nearby vasculature. As an alternative, we decided to proceed with an ESPB with indwelling catheter placement. Unlike a brachial plexus nerve catheter, we anticipated the ESP catheter would provide sensory blockade to not only the right upper extremity but also the proximal shoulder and right chest wall.

The ESPB was performed as follows. The patient was placed in the left lateral decubitus position and given 1 mg midazolam and 50 mcg fentanyl for procedural sedation with standard monitoring. Using aseptic precautions, the skin was anesthetized with 5 mL of 2% lidocaine. A high-frequency linear ultrasound transducer was placed in the longitudinal parasagittal plane at the level of the T1 transverse process. A 9-cm-short bevel needle was inserted through the skin and advanced in-plane toward the T1 transverse process, and 15 mL of 0.5% ropivacaine was injected. We visualized the ESM separate away from the T1 transverse process and LA deposited deep into the ESP muscle. A catheter was then threaded in and left at a depth of 10 cm at the skin. After catheter placement was completed, the catheter was secured and then attached to an epidural catheter pump. The pump was started at a constant infusion rate of 5 mL/hour with 0.2% ropivacaine and a demand bolus of an additional 5 mL every 60 minutes. The patient reported improvement in pain immediately following the block.

**Figure 2:**
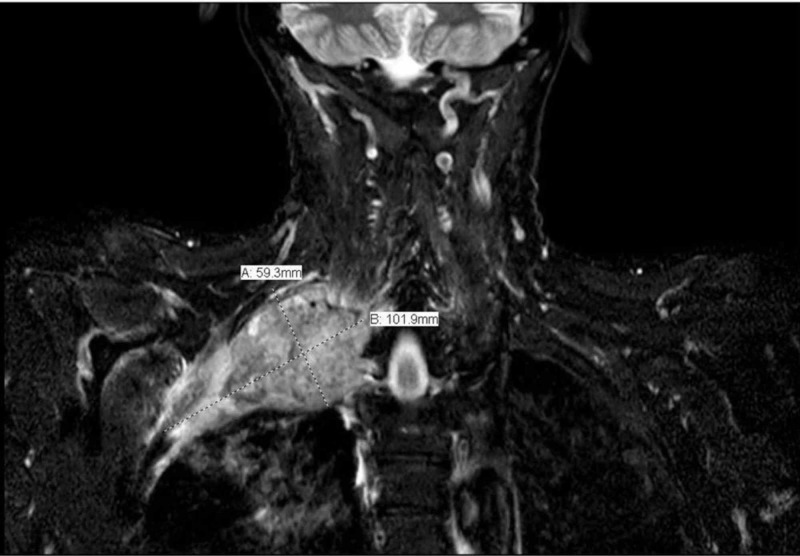
MRI of size, location and extent of right Pancoast tumor MRI, magnetic resonance imaging

On catheter day two, the pump rate was increased to allow the patient to have a demand bolus of 10 mL every 60 minutes while maintaining a constant rate of 5 mL/hour. Over the following three days, the patient experienced an improved range of motion in his right arm, which was previously limited by pain. He endorsed significant pain relief from his right chest wall to his triceps region but with some sparing of his forearm. He stated he felt “more himself in the last couple of days…the pain is out of the way enough”. The patient further reported that his pain had significantly diminished in severity to NRS 4/10. Simultaneously, over this time course, his daily methadone dose decreased by 54.5%, from 110 to 60 mg, and daily OME of opioid usage has decreased (Figure 2).

On catheter day nine, we made the decision to remove the catheter due to ongoing leukocytosis. Unfortunately, after the catheter removal, the patient pain scores increased and opioid consumption increased. The patient was followed up for the next four days as a routine follow-up after the nerve block. There was no complication or residual deficit related to ESP block after catheter removal.

## Discussion

The patient in this case report presented with a wide distribution of pain in the right upper extremity, neck and anterior and lateral chest wall. The extent of Pancoast tumor and bony involvement presented as both somatic and neuropathic pain. There is a lack of robust evidence for the benefit of peripheral nerve blocks and sympathetic blocks to effectively treat neuropathic pain. There is good evidence to support the use of neuraxial blocks to control neuropathic pain, but we had some serious reservations based on the patient’s clinical status and associated adverse effects with neuraxial blocks. The location of the tumor and extent of pain distribution precluded the consideration of pectoral, serratus or brachial plexus blocks. Also, these blocks would not have covered the affected area if used individually.

Because of the severity of his pain, anatomical pain distribution and good response to a prior interventional procedure (cervical epidural steroid injection), we elected to attempt a regional anesthetic technique with the hopes of weaning down his daily opioid requirements. The risks and benefits of an infraclavicular nerve catheter to provide decent analgesia were discussed. After we reviewed prior imaging and assessment with bedside ultrasound, the infraclavicular brachial plexus block was ruled out due to the extent of tumor invasion and its proximity to nearby vasculature. As an alternative, we decided to proceed with an ESPB with indwelling catheter placement.

ESPB is a relatively new regional interfacial plane block that provides somatic and visceral analgesia blocking the dorsal and ventral rami of the spinal nerve along with the rami communicantes that transmit autonomic fibers to and from the sympathetic ganglia. Dorsal ramus divides into medial and lateral cutaneous branches that innervate the skin of the back and the ventral ramus divides into lateral and anterior cutaneous branches that supply lateral and anterior chest wall. ESPB has an extensive craniocaudal spread of LA and is helpful in blocking larger dermatomes. ESPB is technically easy to perform with ultrasound guidance with fewer complications. Unlike an infraclavicular nerve catheter, we hoped the ESP catheter would provide sensory blockade not just to the right upper extremity but also to the neck and right chest wall.

We placed an ESP catheter after injecting initial bolus deep to erector spinae muscle (ESM) at the T1 level. The cadaveric findings and recent case reports have indicated an optimal spread of LA when injected deep to muscle as compared to superficial to muscle. When LA is injected deep into the ESP muscle, it can reach the paravertebral space and intercostal spaces more easily and facilitates sympathetic mediated pain relief as well. The ESM extends along the length of the thoracic and lumbar spine and thus permits a craniocaudal path for LA spread. We expected that our T1 ESP block will cover lower cervical and upper thoracic spinal nerves. The patient had pain relief in most of the areas and it could not be possible with brachial plexus blocks.

The ESP block has been described for treating chronic thoracic pain, acute post-thoracotomy pain and breast surgery, as well as providing abdominal analgesia [19]. There is minimal literature describing the use of ESP block for chronic cancer pain, but it has been successfully reported for palliative pain control in a patient with pleural mesothelioma [20]. The patient in this case report did extremely well with pain control after ESP catheter placement. The patient had severe chronic pain issues with high baseline opioid use on admission. His daily opioid consumption including methadone dose was significantly reduced after ESPB catheter. The ESP catheter was functional for eight days, and patient’s opioid requirements increased back to baseline after catheter removal. There are no specific guidelines for the safe and appropriate duration of ESP catheters; hence, we considered guidelines suggested for peripheral nerve blocks.

The ESP block offers many advantages, which were relevant to our patient. First, it provides coverage of multiple dermatomal levels by either a single injection or catheter. This property clearly favored ESP block over other blocks like pectoral, serratus and brachial plexus blocks.

Second, it provides a simple and safe alternative to paravertebral blocks with minimal or no risk of pneumothorax as the transverse process provides a reliable, consistent sonographic landmark, which serves as a safety backstop for needle advancement. Third, ESP block has minimal epidural spread to cause any significant sympathectomy or hypotension and more hemodynamically stable compared to neuraxial blocks.

## Conclusions

This case report supports that ESP block can be used for the treatment of malignant pain in palliative care settings. This case shows the example of effective analgesia with alternative regional analgesic techniques such as ESP when there are major limitations for nerve blocks and neuraxial blocks. This novel interfascial plane can be used to treat cancer pain with an effective reduction in opioid consumption and it helps improve patient pain relief. The ESP block is one of the newer interfascial blocks with multiple potential applications, but there is limited evidence to support its efficacy. To validate the efficacy of ESP block, rigorous randomized controlled trials are warranted.
